# T cell-engaging CD276xCD3 bispecific antibody for treatment of endometrial cancer

**DOI:** 10.1186/s12967-025-06825-4

**Published:** 2025-07-24

**Authors:** S. M. Greiner, J. Mauermann, M. S. Lutz, I. Hagelstein, A. D. Hartkopf, L. Zekri, G. Jung, H. R. Salih, M. Märklin

**Affiliations:** 1 Clinical Collaboration Unit Translational Immunology, Department of Internal Medicine, University Hospital Tuebingen, Otfried-Mueller-Str. 10, 72076 Tuebingen, Germany; 2 German Cancer Consortium (DKTK), Partner Site Tuebingen, a partnership between DKFZ and University Hospital Tuebingen, 72076 Tuebingen, Germany; 3 Cluster of Excellence iFIT (EXC 2180) ‘Image–Guided and Functionally Instructed Tumor Therapies’, Eberhard Karls University Tuebingen, 72074 Tuebingen, Germany; 4 Department of Obstetrics and Gynecology, University Hospital Tuebingen, 72076 Tuebingen, Germany

**Keywords:** Endometrial cancer, Bispecific antibodies, Immunotherapy

## Abstract

**Background:**

Endometrial cancer ranks among the most prevalent gynecological malignancies, with a notable increase in incidence, especially among women under 40. Although most patients are diagnosed at an early stage and have an excellent prognosis, the outcome for metastatic and recurrent cases remains poor. Current treatment for advanced-stage disease includes chemotherapy, hormonal therapy and checkpoint inhibitors. The clinical response rate to immunotherapy varies depending on the molecular subtype of endometrial carcinoma. Novel immunotherapeutic strategies are needed to improve patient survival, particularly across molecular subtypes. CD276 (cluster of differentiation 276, B7-H3) is emerging as a promising immunotherapy target due to its expression across multiple tumor types. Therapeutic targeting of CD276 may enhance immune cell infiltration into the tumor site by affecting its expression on tumor cells and tumor vasculature, which addresses a critical challenge for the successful treatment of solid tumors.

**Methods:**

We developed a novel, IgG-based CD276xCD3 bispecific antibody termed CC-3, which has demonstrated pronounced preclinical efficacy in stimulating T cell antitumor responses and is presently undergoing evaluation in a Phase I clinical trial (NCT05999396). In this study, CC-3-induced T cell activation and proliferation was analyzed using flow cytometry. We also used a LegendPlex assay to measure the secretion of cytokines and effector molecules induced by CC-3. Finally, these processes culminated in target cell lysis which was analyzed using a flow cytometry-based cytotoxicity assay.

**Results:**

CD276 is abundantly expressed in endometrial cancer. Treatment with CC-3 activated T cells, stimulated degranulation, and induced the secretion of cytokines and effector molecules, demonstrating CC-3-mediated T cell reactivity against endometrial cancer cells. Furthermore, CC-3 promoted robust T cell proliferation and memory T cell subset formation, culminating in potent target cell lysis.

**Conclusion:**

Overall, our findings highlight the potential of CC-3 for clinical evaluation as a therapeutic option for patients with endometrial cancer.

**Supplementary Information:**

The online version contains supplementary material available at 10.1186/s12967-025-06825-4.

## Introduction

Endometrial cancer is the second most prevalent gynecological malignancy with poor outcomes for metastatic and recurrent disease and is classified into four molecular subtypes identified by TCGA (The Cancer Genome Atlas) that differ in biology, prognosis and appropriate therapy [1]. POLE (gene encoding DNA polymerase epsilon) ultramutated and microsatellite instability hypermutated (MSI-H) subtypes carry a high mutational burden and respond well to immunotherapy. The copy number low subtype has a good prognosis and standard treatment may be sufficient, whereas copy number high subtypes often require aggressive treatment [2]. Recently, a more pragmatic clinically applicable classification was introduced to distinguish cases of POLEmut, mismatch repair deficient (MMRd), TP53 mutated subtype and no specific molecular profile with normal p53 expression/p53wt [3]. The current standard treatment for endometrial cancer typically involves a combination of surgery, radiation therapy, and sometimes chemotherapy or hormonal therapy, depending on the stage and molecular characteristics of the tumor [1]. Several clinical trials have shown positive results for the combinations of immune checkpoint inhibitors and chemotherapy. The combination of atezolizumab with chemotherapy in the AtTEnd trial increased progression free survival of patients, especially in those with MMRd carcinomas [4]. Similar results were obtained by the addition of dostarlimab in RUBY trial or pembrolizumab in NRG-GY018 trial [5, 6]. Chemotherapy-free regimens such as lenvatinib plus pembrolizumab or addition of immunotherapeutics to chemotherapy such as durvalumab plus carboplatin/paclitaxel are approved for relapse after chemotherapy and have shown benefit in progression-free survival [7, 8]. In conclusion, new immunotherapeutic approaches are needed for patients who do not respond to checkpoint inhibition, as well as for those who experience limited benefit from treatment in the absence of MMRd.

One promising target for new immunotherapeutic approaches is CD276 (B7-H3), which is highly expressed in multiple tumor types. Its expression not only on tumor cells but also on the tumor vasculature has been shown to facilitate infiltration of immune effector cells into the tumor site, which is a prerequisite for efficacy of T cell-based therapies in solid tumors [9]. CD276 expression in endometrial cancer is associated with higher tumor grade, shorter overall survival (OS) and less tumor infiltrating lymphocytes [10, 11]. Recently, we have developed an IgG-based CD276xCD3 bispecific antibody (bsAb) termed CC-3 that showed preclinical efficacy in stimulating T cell responses against various gastrointestinal cancer types, sarcoma and breast cancer [9, 12–14]. Furthermore, CC-3 is presently undergoing evaluation in a phase I clinical trial in colorectal and breast cancer as well as sarcoma (NCT05999396). Here we evaluated the therapeutic potential of CC-3 for treatment of endometrial cancer.

## Materials and methods

### Generation and purification of bispecific antibodies

Generation of CC-3 and its isotype control (MOPCxCD3) has been described previously [12, 14]. The constructs were produced in ExpiCHO cells (Gibco, Carlsbad, CA, USA) and purified from culture supernatant by affinity chromatography on Mabselect affinity columns (GE Healthcare, Munich, Germany) followed by analytical and preparative size exclusion chromatography on Superdex S200 Increase 10/300GL and HiLoad 16/60 columns (GE Healthcare). EndoZyme II (BioMerieux, Marcy-l’Étoile, France) was used according to the manufacturer’s instructions to measure endotoxin levels, which were less than 0.5 EU/ml.

### TCGA database analysis of B7-H3 gene expression

RNA sequencing data on relative B7-H3 expression in endometrial cancer tissues from the Cancer Genome Atlas database and the Genotype-Tissue Expression project were analyzed using the Gene Expression Profiling Interactive Analysis (GEPIA) web server.

### Cell lines and primary cells

The endometrial cancer cell lines An3CA, Ishikawa and Hec1a were used. An3CA was derived from a lymph node metastasis, Ishikawa from a well-differentiated endometrial adenocarcinoma and Hec1a was isolated from a patient with early-stage endometrial cancer [15–17]. Molecular characterization of endometrial cancer cell lines in TCGA subtypes has not yet been established. In addition, the B7-H3 negative HL-60 cell line was used. Cells were routinely tested for mycoplasma contamination every month. Peripheral blood mononuclear cells (PBMCs) from healthy donors were obtained after written informed consent after ethical approval of the study (reference number 13/2007V). PBMCs were isolated by density gradient centrifugation (Biocoll; Biochrom, Berlin, Germany).

### Flow cytometry

To analyze CD276 surface expression, endometrial cancer cells were incubated with CD276 monoclonal antibody clone 7C4, which is also contained in the targeting moiety of CC-3, or the corresponding isotype control followed by a goat anti-mouse PE conjugate (Dako, Glostrup, Denmark). CD25-PE, CD69-PE, CD107a-PE (BD Pharmingen) and CD4-APC, CD8-FITC, CD62L-PB and CD45RO-PeCy7 (BioLegend, San Diego, CA) fluorescent conjugates were used to determine T cell activation, degranulation and proliferation. 7-aminoactinomycin D (7-AAD) (Biolegend) was used to distinguish live from dead cells. Measurements were performed using a Fortessa cytometer (BD Biosciences, San Diego, CA) and data were analyzed using FlowJo software (FlowJo LCC, Ashland, OR).

### Antigen shift assay

Endometrial cancer cells were incubated with CC-3 or MOPCxCD3 isotype control at the indicated concentrations for 48 h. To measure CD276 expression, cells were washed and stained with 10 µg/ml anti-CD276 antibody (clone 8H8), followed by an anti-mouse PE conjugate and analyzed by flow cytometry.

### T cell activation and degranulation assays

Activation and degranulation in the presence of target cells was determined in a culture of tumor cells with PBMCs from healthy donors (effector to target (E:T) 4:1) in the presence or absence of CC-3 or isotype control (0.25 nM each). To analyze T cell degranulation and activation, CD107a, CD69 and CD25 expression was determined after 4 h, 24 h and 72 h respectively. Analysis was performed using flow cytometry.

### T cell proliferation and differentiation assays

To determine long-term proliferation of T cells, PBMCs were loaded with 2 μM CellTrace™ Violet cell proliferation dye (Thermo Fisher Scientific) and incubated with tumor cells (E:T 4:1) and CC-3 or isotype control (0.25 nM each). After 72 h, the cells were reexposed to fresh target cells and the respective treatment. Another 96 h later, the proliferation of CD4^+^ and CD8^+^ T cells was analyzed by cell count in flow cytometry. For T cell subset analysis, the same protocol was followed until day 7 when T cell subpopulations were identified by flow cytometric analysis for the expression of CD4, CD8, CD45RO and CD62L.

### Analysis of effector molecules and cytokine secretion

Supernatants of PBMCs from healthy donors cultured with tumor cells (E:T 4:1) in the presence or absence of CC-3 or isotype control (0.25 nM each), were collected after 24 h. The supernatants were analyzed for immunomodulatory cytokines (interleukin-2 (IL-2), tumor necrosis factor (TNF), interferon-γ (IFN-γ)) and effector molecules (granzyme A, granzyme B, perforin and granulysin) using LEGENDplex assays (BioLegend).

### Cytotoxicity assays

For flow cytometric analysis of target cell lysis, tumor cells were labeled with 2 μM CellTrace™ Violet (Thermo Fisher Scientific) and cultured with PBMCs from healthy donors (E:T 4:1) in the presence or absence of CC-3 or isotype control (0.25 nM each) for 72 h. Standard calibration latex beads (Sigma-Aldrich, St. Louis, MO) were used to determine the number of target cells that vanished from the culture and to ensure analysis of equal test volumes. The lysis of endometrial cancer cells was analyzed after 72 h using 7-AAD to distinguish between live and dead cells. The same protocol was used for long-term cytotoxicity analysis and measurements were performed using the xCELLigence RTCA system (Roche Applied Science, Penzberg, Germany).

### Statistics

All statistical analyses were performed using GraphPad Prism (version 8.1.0). The data were tested for normality using the Shapiro–Wilk test. Data that did not meet the assumption of normal distribution, were assessed for statistical significance between different conditions using the nonparametric Kruskal–Wallis test. If the data met the assumption of normal distribution, an ANOVA was performed. Data is shown as mean ± SEM. The significance level was set at P < 0.05.

## Results

### CD276 expression in endometrial cancer cell lines

**Fig. 1:**
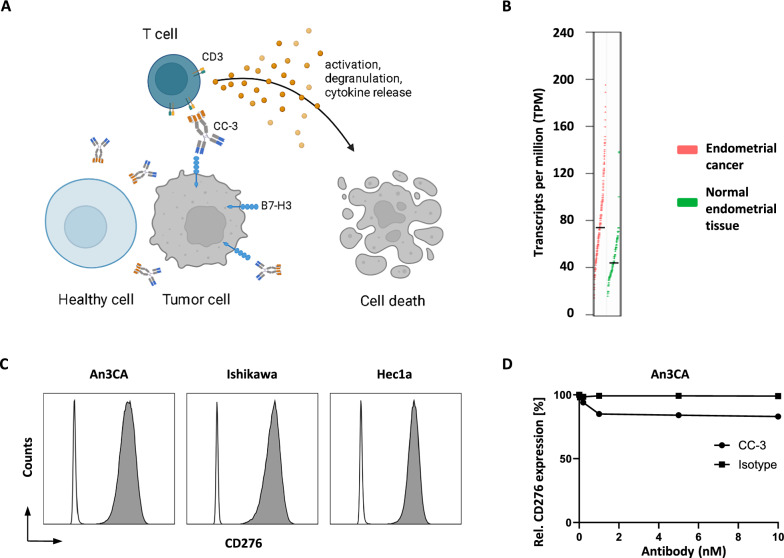
Characterization of CD276 expression and CC-3 binding in endometrial cancer cell lines. **A** Schematic representation and mechanism of action of CC-3. The graph was generated by BioRender (BioRender.com, Toronto, Canada). **B** CD276 gene expression profile in endometrial cancer (n = 174) and normal endometrial tissue (n = 91). Data were analyzed using the Gene Expression Profiling Interactive Analysis (GEPIA) web server. GEPIA uses RNA sequencing expression data from TCGA database and the GTEx project. **C** The indicated cancer cell lines were stained with a murine CD276 monoclonal antibody (clone 7C4) followed by an anti-mouse PE conjugate and analyzed by flow cytometry. CD276 expression on endometrial cancer cell lines An3Ca, Ishikawa and Hec1a is shown (shaded peaks, anti-B7H3; open peaks, control). **D** An3Ca cells were incubated with the indicated concentrations of CC-3 or MOPCxCD3 isotype control for 48 h, then the expression of CC-3 was analyzed by flow cytometry using a murine monoclonal antibody CD276 (clone 8H8) followed by an anti-mouse PE conjugate

Figure 1A provides a schematic representation of the mechanism of action of CC-3. CC-3 binds to the tumor antigen CD276 and to CD3 on T cells, leading to T cell activation, proliferation, differentiation, and ultimately lysis of target cells. To determine the potential of CC-3 in endometrial cancer, as a first step CD276 mRNA expression was analyzed in a TCGA dataset of 174 endometrial cancer samples and compared with 91 healthy tissue samples, showing higher expression in cancer samples (Fig. 1B). We characterized CD276 surface expression on different endometrial cancer cell lines. For the analysis we used the CD276 monoclonal antibody clone 7C4, which is also contained in the targeting moiety of CC-3. All cell lines showed substantial CD276 expression, as shown by the flow cytometric analysis (Fig. 1C). Next, we analyzed whether CD276 expression was stable after exposure to CC-3 or isotype control for 72 h. No significant alteration in surface expression levels over time were detected (Fig. 1D).

### CC-3 induces T cell activation against endometrial cancer cell lines

**Fig. 2:**
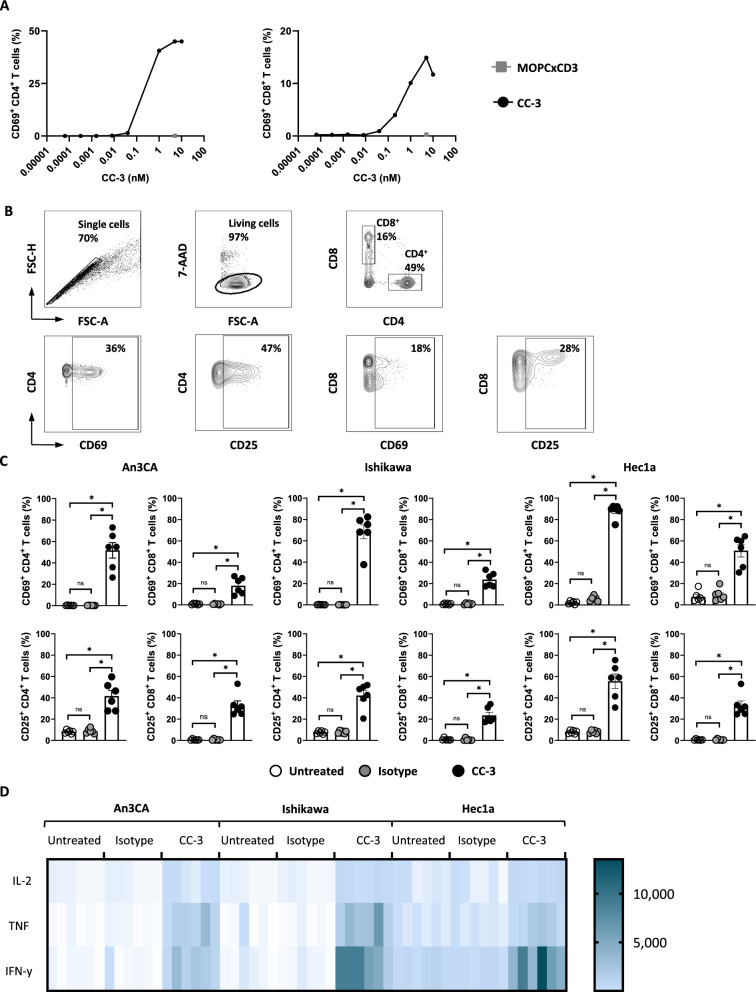
Induction of T cell activation against endometrial cancer cell lines by CC-3. **A** PBMCs from healthy donors were incubated with the indicated tumor cell line (E:T 4:1) with increasing concentrations of CC-3 as indicated or the isotype control at 5nM. Activation of CD4^+^ and CD8^+^ T cells was determined by flow cytometric analysis for CD69 expression after 24 h. Exemplary data with PBMCs from one donor is shown. **B** An exemplary gating strategy is shown: singlets, viable (7-AAD-), CD4^+^CD8^+^ T cells, CD69 or CD25 expression on CD4^+^ and CD8^+^ T cells, respectively. **C** Activation of CD4^+^ and CD8^+^ T cells was determined by flow cytometric analysis for CD69 expression at 24 h. Late activation was determined by CD25 expression at 72 h. Combined data obtained with PBMCs from six independent donors are shown. **D** IL-2, TNF and IFN-γ levels in culture supernatants were measured after 24 h using LEGENDplex assays. Combined data obtained with PBMCs from six independent donors are shown

To determine the optimal concentration of CC-3 for subsequent experiments, we performed a dose titration of CC-3 with Ishikawa cells and PBMCs to assess T cell activation. The results demonstrated potent effects already at low concentrations, with saturated activity between 0.1 and 1 nM. Importantly, no unspecific T cell activation was observed with an isotype control targeting an irrelevant antigen (Fig. 2A). Based on these findings, a concentration of 0.25 nM CC-3 was selected for all further experiments. Next, we cultured PBMCs from healthy donors with endometrial cancer cell lines and CC-3, isotype control or without treatment and determined the potential of CC-3 to induce T cell activity using different endometrial cancer cell lines. Activation of CD4^+^ and CD8^+^ T cells was determined by analysis of CD69 and CD25 after 24 h and 72 h, respectively. Treatment with CC-3 induced significantly higher expression of CD69 and CD25 compared to the isotype and untreated control, confirming the target cell-restricted mode of action of T cell activation by CC-3 (Fig. 2B, C). In line with these results, analysis of culture supernatants by LEGENDPlex assays showed a significant increase in IL-2, TNF, and IFN-γ secretion after treatment with CC-3, whereas no effect was observed with the isotype control. Cytokine concentrations are shown in heat blots for every donor and treatment using three different endometrial cancer cell lines (Fig. 2D, Figure S2).

### CC-3 induces T cell degranulation

**Fig. 3:**
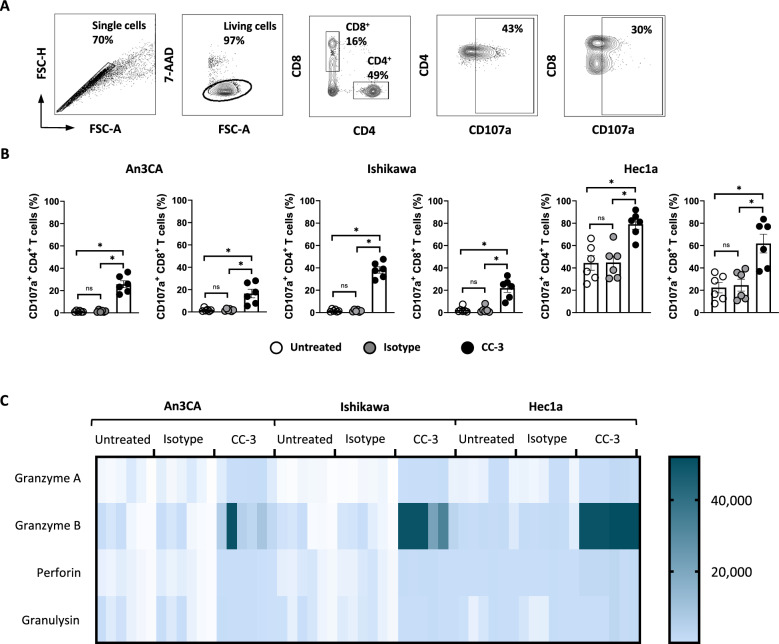
Treatment with CC-3 induces T cell degranulation and release of effector molecules. PBMCs from healthy donors were incubated with the indicated tumor cell lines (E:T 4:1) in the presence or absence of CC-3 or isotype control at 0.25 nM. **A** An exemplary gating strategy is shown: singlets, viable (7-AAD-), CD4^+^CD8^+^ T cells, CD107a expression on CD4^+^ and CD8^+^ T cells respectively. **B** Degranulation of CD4^+^ and CD8^+^ T cells was determined by CD107a expression after 24 h. Combined data obtained with PBMCs from 6 independent donors are shown. **C** Granzyme A, granzyme B, perforin and granulysin levels in culture supernatants were measured after 24 h using Legendplex assays. The combined data obtained from PBMCs from six independent donors are shown

To analyze T cell reactivity against endometrial cancer cells after CC-3 treatment, degranulation of CD4^+^ and CD8^+^ T cells and release of effector molecules were determined. PBMCs from healthy donors were cultured with endometrial cancer cell lines and CC-3, the isotype control or without treatment, and the expression of CD107a as surrogate marker for degranulation was analyzed after 4 h. CC-3 potently stimulated T cell degranulation as revealed by a pronounced expression of CD107a after treatment with CC-3 whereas no effect was observed with the isotype control, again confirming the target restricted activity of our construct (Fig. 3A, B). In line with this, analysis of culture supernatants with LEGENDPlex assays showed significantly higher release of the effector molecules granzyme A, granzyme B, perforin and granulysin after CC-3 treatment compared to the isotype control (Fig. 3C, Figure S2).

### CC-3 induces T cell proliferation and memory subset formation

**Fig. 4:**
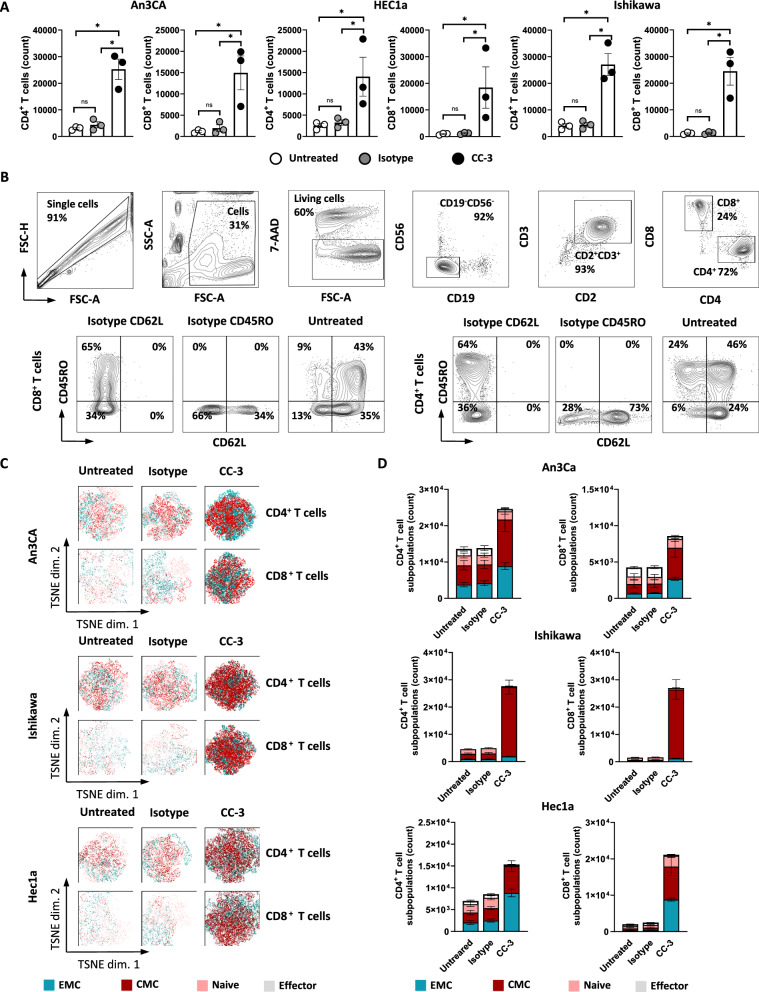
T cell proliferation and memory T cell populations induced by CC-3. **A** PBMCs from healthy donors (n = 3) were labeled with CellTrace™ Violet cell dye and incubated with or without isotype control or CC-3 (0.25 nM each) in the presence of Ishikawa, An3CA or Hec1a cells (E:T 4:1). After 72 h, PBMCs were re-exposed to fresh target cells and the respective treatment for another 72 h. On day 6, proliferation was determined by flow cytometry. Pooled data of 3 independent PBMCs donors are shown. **B, C** PBMCs from healthy donors (n = 3) were incubated with or without isotype control or CC-3 (0.25 nM each) in the presence of Ishikawa, An3CA or Hec1a cells (E:T 1:1). After 72 h, the cells were reexposed to fresh target cells and the respective treatment. On day 6, subpopulations of CD4^+^ and CD8^+^ T cells were determined by flow cytometric analysis. Effector T cells (Effector) were defined as CD62L^−^CD45RO^−^, naive T cells (Naive) as CD62L^+^CD45RO^−^, effector memory T cells (EMC) as CD62L^−^CD45RO^+^ and central memory T cells (CMC) as CD62L^+^CD45RO^+^. **B** An exemplary gating strategy is shown: singlets, viable (7-AAD-), CD4^+^CD8^+^ T cells, CD62L and CD45RO expression on CD4^+^ and CD8^+^ T cells respectively **(C)** representative t-distributed stochastic neighbor embedding (tSNE) plots and **(D)** pooled data are shown

T cell proliferation is a critical requirement to effectively combat high tumor burden. To analyze whether and how CC-3 induced T cell proliferation, we labeled PBMCs of healthy donors using CellTrace™ Violet and analyzed CD4^+^ and CD8^+^ T cell counts in cocultures with endometrial tumor cells upon exposure to CC-3 or control by flow cytometry (Fig. 4A–D). In samples treated with CC-3, the number of T cells increased significantly compared to untreated controls, whereas the isotype control had no effect (Fig. 4A). To asses differentiation into memory T cell subsets, we used flow cytometry to measure CD62L and CD45RO expression on CD4^+^ and CD8^+^ T cells, respectively. We found that treatment with CC-3 induced a significant expansion of effector memory (ECM) and central memory (CMC) T cells (Fig. 4C, D), and again this effect occurred in a target-restricted manner, as isotype control had no effect. Off note, analysis of CD3 showed that effector T cells expressed higher levels of CD3 compared to naïve T cells. This may be of clinical importance since CC-3 could engage effector T cells via CD3 binding more effectively (Figure S3).

### CC-3 induces lysis of endometrial cancer cell lines

**Fig. 5:**
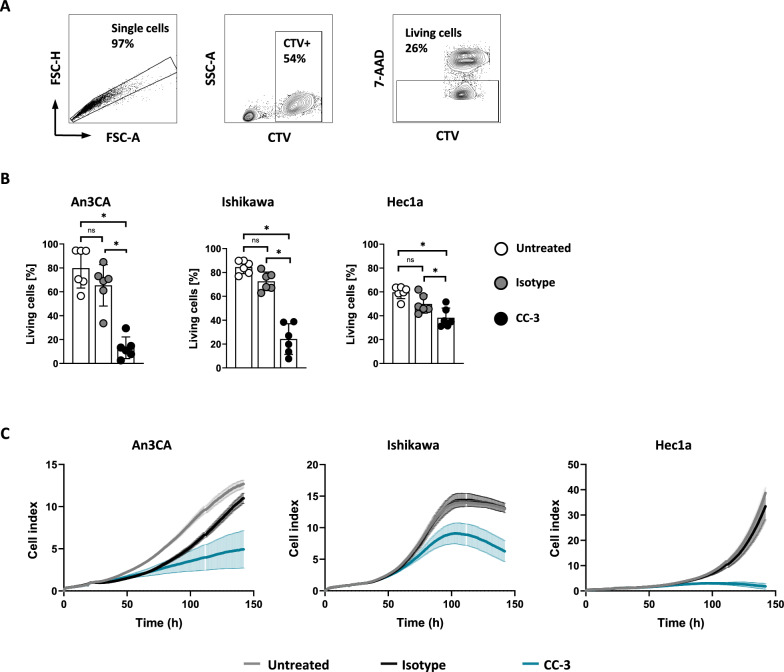
CC-3 induces tumor cell lysis of endometrial cancer cells. PBMCs from healthy donors were incubated with the indicated target cells at an E:T ratio of 4:1 in the presence or absence of isotype control or CC-3 (0.25 nM each). **A, B** Lysis of An3Ca, Ishikawa and Hec1a cells was determined by flow cytometry-based lysis assay after 72 h. An exemplary gating strategy **(A)** and combined data obtained from PBMCs from six independent donors are shown **(B)**. **C** The long-term cytotoxic effects of PBMCs from healthy donors (n = 4) on An3Ca, Ishikawa and Hec1a cells were determined using the xCELLigence system

To analyze whether CC-3 treatment induced T cell activation, differentiation and proliferation is mirrored by lysis of endometrial cancer cells PBMCs from healthy donors were co-cultured with tumor target cells and treated with CC-3, isotype control, or were left untreated. Cancer cell lysis was analyzed using a flow cytometry-based lysis assay, which revealed that after 72 h, CC-3 significantly induced endometrial cancer cell lysis. In line with our previous results, target cell lysis occurred in a target cell-restricted manner, as the isotype had no effect (Fig. 5A, B). Of note, treatment of CD276-negative HL-60 cells with the isotype control MOPCxCD3 or CC-3 further supports target cell restriction, confirming that the activity of our construct is strictly restricted to the target antigen (Supplementary figure S1). Most importantly, we also observed pronounced therapeutic efficacy of CC-3 in long-term lysis assays using the xCELLigence system, which showed efficacy of treatment over an extended time period of ~ 140 h (Fig. 5C).

## Discussion

In recent years, immunotherapeutic approaches have gained increasing importance for the treatment of endometrial cancer. The RUBY trial evaluated dostarlimab (anti-PD-1 therapy) in combination with carboplatin and paclitaxel. It demonstrated a substantial survival benefit for patients with MMRd tumors and some benefit in proficient (MMRp) ones [5]. In patients with advanced or recurrent endometrial cancer, the addition of pembrolizumab to standard chemotherapy resulted in significantly longer progression-free survival. Although pembrolizumab was formally effective in both MMRd and MMRp, its efficacy was still highly dependent on MMR status [6]. The DUO-E phase III trial assessed the addition of immunotherapy (durvalumab, an anti-PD-L1 immunotherapy) and targeted therapy (poly (ADP-ribose) polymerase (PARP) inhibitor olaparib) to standard chemotherapy (carboplatin/paclitaxel) in advanced or recurrent endometrial cancer. The results revealed a reduction of disease progression of 29% with durvalumab and a 45% reduction with combination of durvalumab and olaparib. Notably, the benefit of adding olaparib was more pronounced in MMRp group [8]. The addition of immunotherapy to chemotherapy alone significantly improved in progression-free survival. This benefit was less pronounced in the MMRp group than in the MMRd group (HR = 0.33 vs HR = 0.74) [18]. Novel immunotherapeutic approaches are needed to further improve patient outcomes regardless of molecular subtype, ideally reducing adverse events and improving the quality of life for patients with advanced and recurrent disease. To address this medical need, and as TCGA data indicated high expression of the target antigen CD276 on tumor cells, we here evaluated the efficacy of our bsAb CC-3 targeting CD276 and CD3 as treatment option for endometrial cancer.

The choice of CD276 as a target is based on the fact that it is expressed on both tumor cells and tumor vasculature across various tumor types. CD276 expression in tumor vasculature may facilitate T cell infiltration into the tumor, thereby overcoming a limiting factor for successful T cell-based immunotherapy in solid cancers. In addition, high CD276 expression is associated with poorer prognosis in various cancer entities [19–22], e.g. higher tumor grade, shorter OS, and a lower presence of tumor-infiltrating lymphocytes [10, 11]. Based on these data, we reasoned that targeting CD276 to improve its influx into the tumor site and achieve subsequent tumor cell killing in endometrial cancer with CC-3 would constitute a promising approach.

We found that CD276 was highly expressed in three endometrial cancer cell lines. Treatment with CC-3 potently induced T cell activation, degranulation, proliferation and differentiation into memory T cell subtypes, which are critical for clinical efficacy. In addition, CC-3 induced secretion of antitumor cytokines (IFN-γ, TNF, IL-2) and effector molecules (granzymes, perforin, and granulysin), which are known to promote tumor cell death, and also induced potent and long-lasting tumor cell lysis by the activated T cells. These results expand data we recently reported for other solid tumor entities and identify endometrial cancer as an additional disease entity where CC-3 treatment may prove beneficial [9, 12–14]. Currently, CC-3 is being evaluated in a first-in-human trial (NCT05999396) that enrolls patients with colorectal cancer, sarcoma, and breast cancer. This trial will reveal whether CC-3 exhibits antitumor potential, as suggested by preclinical studies, when tested in real life where critical factors such as the tumor microenvironment, T cell infiltration, tumor vasculature, and T cell exhaustion come into play. Several clinical trials are currently evaluating the efficacy of bsAb in endometrial cancer (NCT06515613, NCT03564340, NCT06522828, NCT05032040, NCT03752398 and NCT03849469). Solotimab, a bsAb targeting EpCAM and CD3, showed effective T cell activation and cancer cell lysis in vitro [23]. This makes bispecific antibodies an interesting concept for new immunotherapeutic approaches in the therapy of endometrial cancer, even if broad expression of its target antigen requires careful evaluation of potential side effects due to “on target off tumor” T cell activation.

The results of our trial evaluating CC-3 in other cancer entities, together with the data on its preclinical efficacy in endometrial cancer provided here, may pave the way for the development of a promising new immunotherapeutic approach to be clinically evaluated in patients with endometrial cancer.

## Supplementary Information

Additional file 1

## Data Availability

The datasets supporting the conclusions of this article are available upon reasonable request to our corresponding author HRS.

## Abbreviations

7-AAD: 7-Aminoactinomycin D
bsAb: Bispecific antibody
CD: Cluster of differentiation
E:T: Effector to target
FSC: Forward scatter
IFN-γ: Interferon γ
IL: Interleukin
MMR: Mismatch repair
MMRd: Mismatch repair deficient
MMRp: Mismatch repair proficient
MSI-H: Microsatellite instability hypermutated
OS: Overall survival
PBMCs: Peripheral blood mononuclear cells
POLE: Gene encoding the DNA polymerase epsilon
SD: Standard deviation
SSC: Side scatter
TCGA: The Cancer Genome Atlas
TNF: Tumor necrosis factor
