# Supplementary material for: T cell-engaging CD276xCD3 bispecific antibody for treatment of endometrial cancer
Source: J Transl Med. 2025 Jul 24;23:825. doi: 10.1186/s12967-025-06825-4 (PMC12291243; doi:10.1186/s12967-025-06825-4)
Supplement: Supplementary file 1 — Additional file 1 [file 12967_2025_6825_MOESM1_ESM.pptx]

## Slide 1
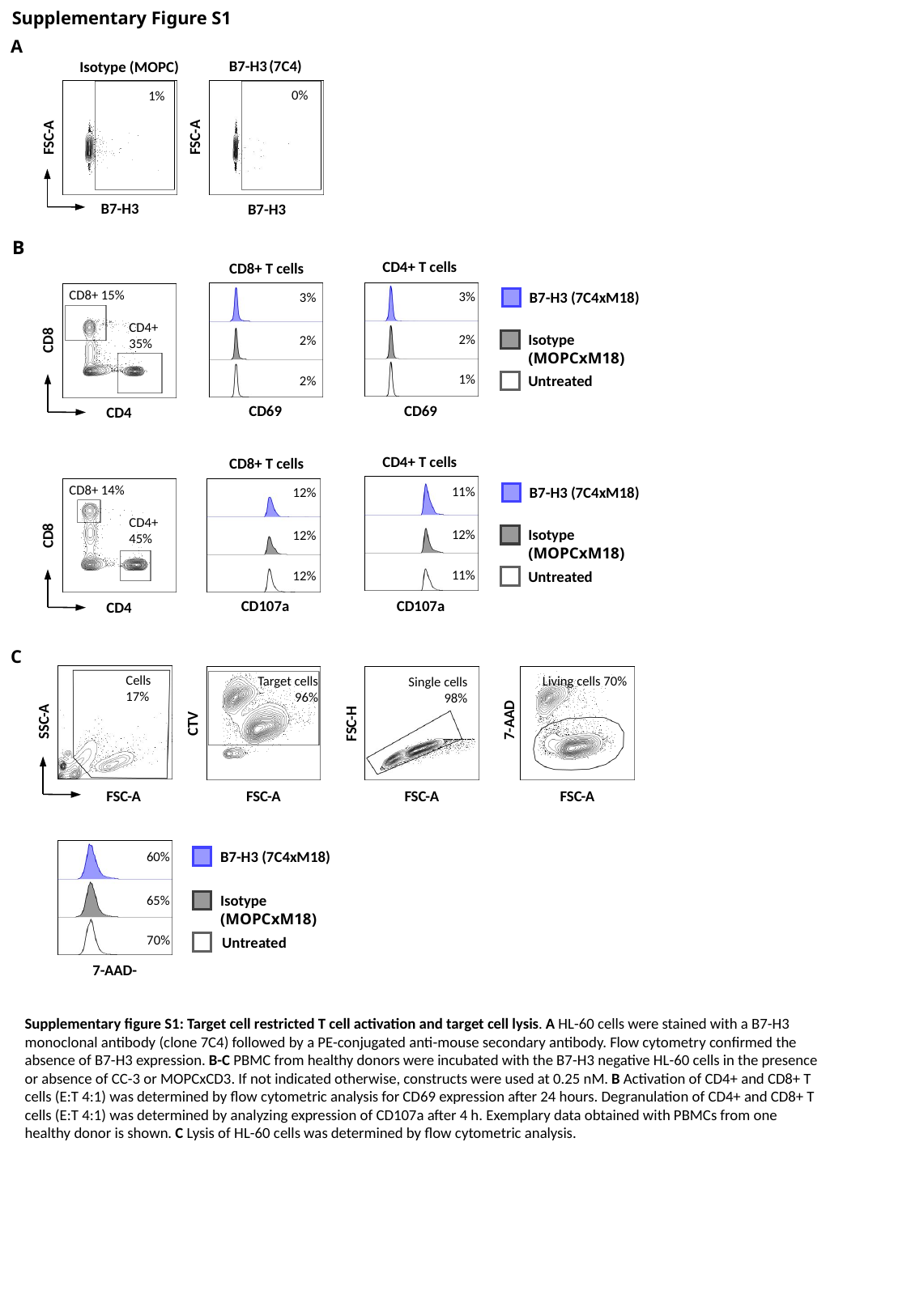

Supplementary Figure S1
A
B7-H3 (7C4)
Isotype (MOPC)
0%
1%
FSC-A
FSC-A
B7-H3
B7-H3
B
CD4+ T cells
CD8+ T cells
CD8+ 15%
3%
B7-H3 (7C4xM18)
3%
CD4+ 35%
Isotype (MOPCxM18)
2%
CD8
2%
1%
Untreated
2%
CD69
CD69
CD4
CD4+ T cells
CD8+ T cells
CD8+ 14%
11%
B7-H3 (7C4xM18)
12%
CD4+ 45%
Isotype (MOPCxM18)
12%
CD8
12%
11%
Untreated
12%
CD107a
CD107a
CD4
C
Cells 17%
SSC-A
FSC-A
Target cells 96%
CTV
FSC-A
Single cells 98%
FSC-H
FSC-A
Living cells 70%
7-AAD
FSC-A
B7-H3 (7C4xM18)
60%
Isotype (MOPCxM18)
65%
70%
Untreated
7-AAD-
Supplementary figure S1: Target cell restricted T cell activation and target cell lysis. A HL-60 cells were stained with a B7-H3 monoclonal antibody (clone 7C4) followed by a PE-conjugated anti-mouse secondary antibody. Flow cytometry confirmed the absence of B7-H3 expression. B-C PBMC from healthy donors were incubated with the B7-H3 negative HL-60 cells in the presence or absence of CC-3 or MOPCxCD3. If not indicated otherwise, constructs were used at 0.25 nM. B Activation of CD4+ and CD8+ T cells (E:T 4:1) was determined by flow cytometric analysis for CD69 expression after 24 hours. Degranulation of CD4+ and CD8+ T cells (E:T 4:1) was determined by analyzing expression of CD107a after 4 h. Exemplary data obtained with PBMCs from one healthy donor is shown. C Lysis of HL-60 cells was determined by flow cytometric analysis.

## Slide 2
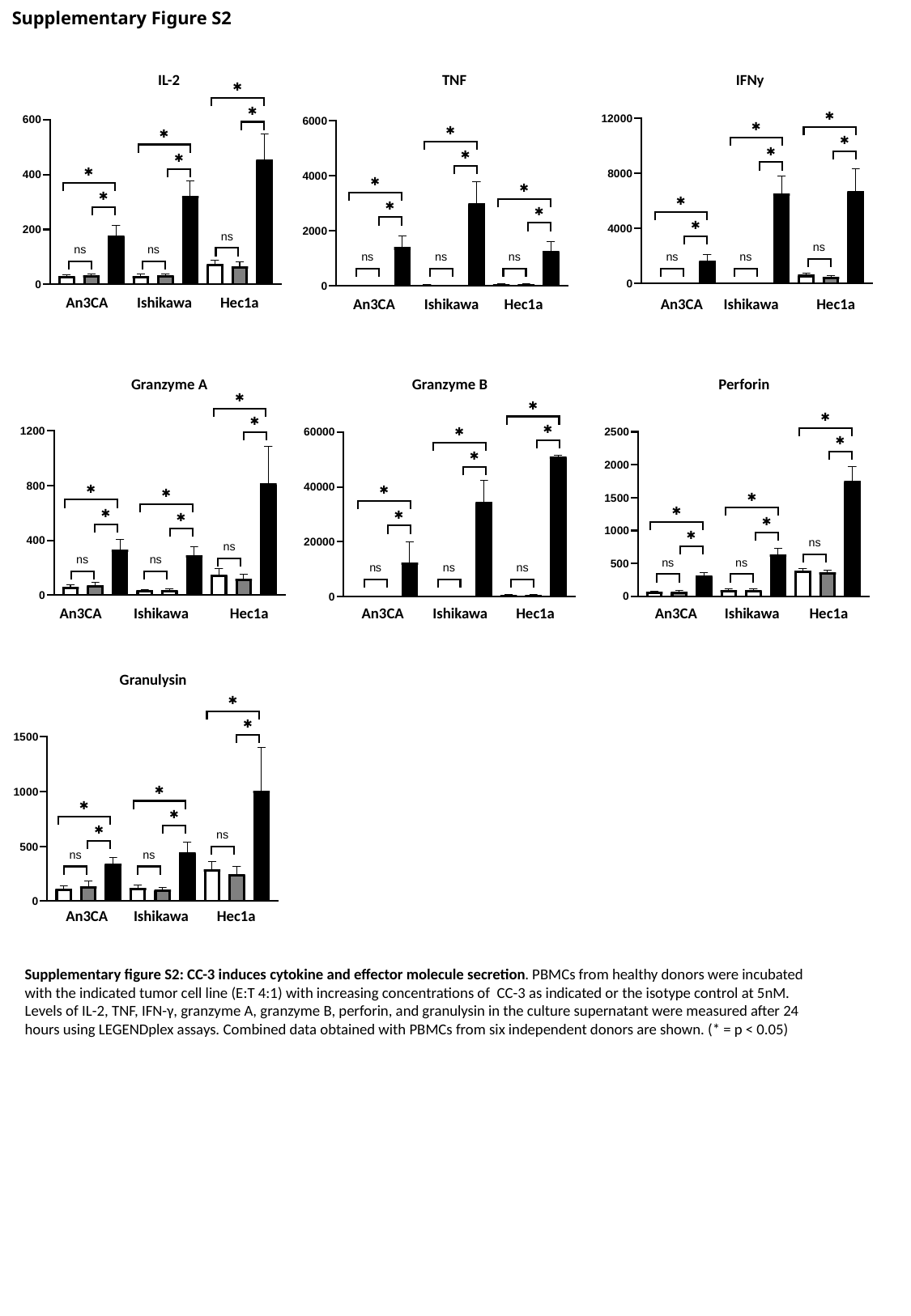

Supplementary Figure S2
IL-2
TNF
IFNy
An3CA
Ishikawa
Hec1a
An3CA
Ishikawa
Hec1a
An3CA
Ishikawa
Hec1a
Granzyme A
Granzyme B
Perforin
An3CA
Ishikawa
Hec1a
An3CA
Ishikawa
Hec1a
An3CA
Ishikawa
Hec1a
Granulysin
An3CA
Ishikawa
Hec1a
Supplementary figure S2: CC-3 induces cytokine and effector molecule secretion. PBMCs from healthy donors were incubated with the indicated tumor cell line (E:T 4:1) with increasing concentrations of CC-3 as indicated or the isotype control at 5nM. Levels of IL-2, TNF, IFN-γ, granzyme A, granzyme B, perforin, and granulysin in the culture supernatant were measured after 24 hours using LEGENDplex assays. Combined data obtained with PBMCs from six independent donors are shown. (* = p < 0.05)

## Slide 3
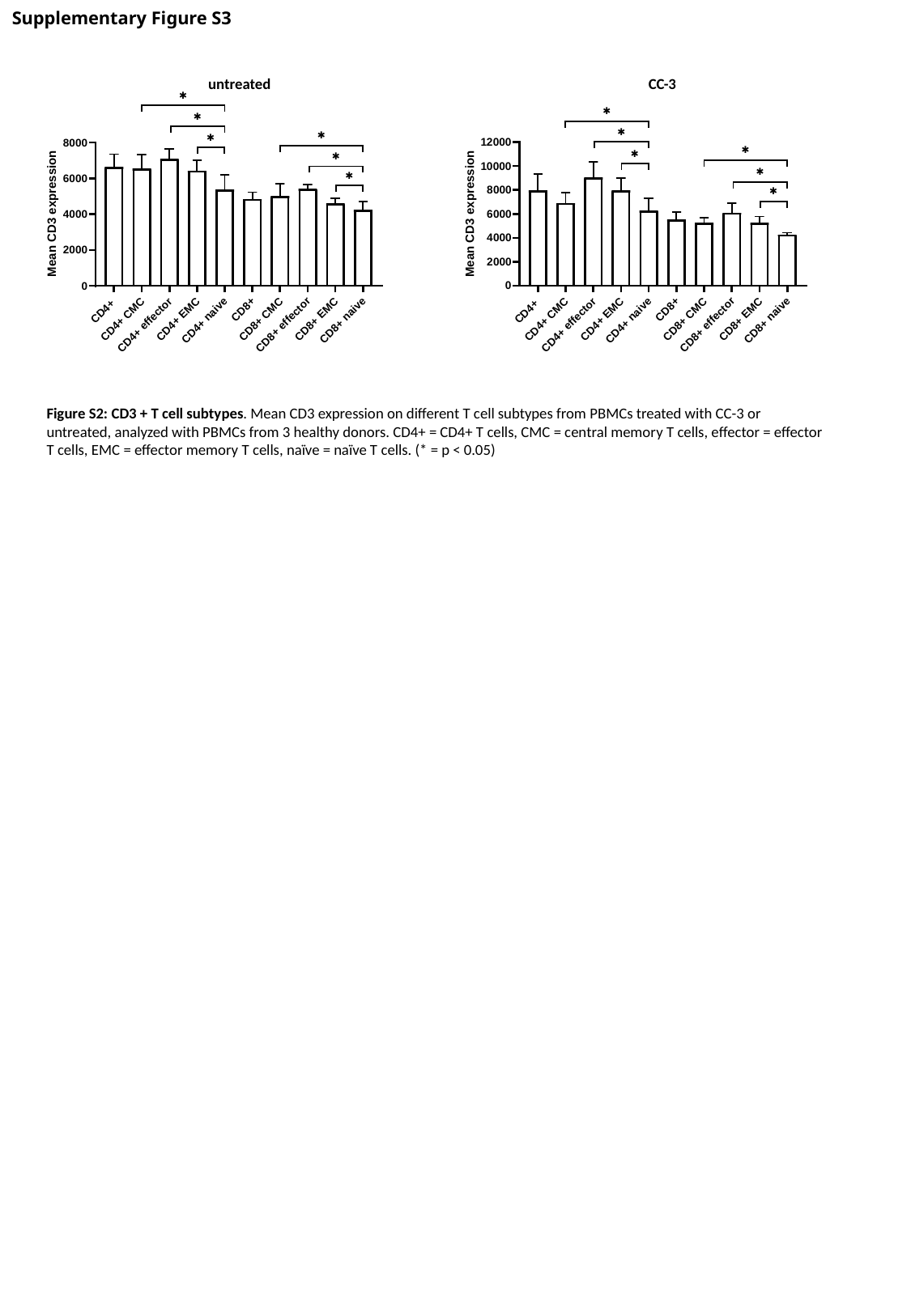

Supplementary Figure S3
untreated
CC-3
Figure S2: CD3 + T cell subtypes. Mean CD3 expression on different T cell subtypes from PBMCs treated with CC-3 or untreated, analyzed with PBMCs from 3 healthy donors. CD4+ = CD4+ T cells, CMC = central memory T cells, effector = effector T cells, EMC = effector memory T cells, naïve = naïve T cells. (* = p < 0.05)
